# Estrogen Protects Neurotransmission Transcriptome During Status Epilepticus

**DOI:** 10.3389/fnins.2018.00332

**Published:** 2018-06-20

**Authors:** Dumitru A. Iacobas, Sanda Iacobas, Nino Nebieridze, Libor Velíšek, Jana Velíšková

**Affiliations:** ^1^Center for Computational Systems Biology, Prairie View A&M University, Prairie View, TX, United States; ^2^DP Purpura Department of Neuroscience, Albert Einstein College of Medicine, New York, NY, United States; ^3^Department of Pathology, New York Medical College, Valhalla, NY, United States; ^4^Department of Cell Biology & Anatomy, New York Medical College, Valhalla, NY, United States; ^5^Department of Pediatrics, New York Medical College, Valhalla, NY, United States; ^6^Department of Neurology, New York Medical College, Valhalla, NY, United States; ^7^Department of Obstetrics & Gynecology, New York Medical College, Valhalla, NY, United States

**Keywords:** beta-estradiol, cholinergic synapse, dentate gyrus, dopaminergic synapse, GABAergic synapse, glutamatergic synapse, jun oncogene, serotonergic synapse

## Abstract

Women with epilepsy commonly have premature onset of menopause. The decrease in estrogen levels is associated with increased occurrence of neurodegenerative processes and cognitive decline. Previously, we found that estradiol (E2) replacement in ovariectomized (OVX) female rats significantly reduced the seizure-related damage in the sensitive hilar region of hippocampal dentate gyrus (DG). However, the complex mechanisms by which E2 empowers the genomic fabrics of neurotransmission to resist damaging effects of status epilepticus (SE) are still unclear. We determined the protective effects of the estradiol replacement against kainic acid-induced SE-associated transcriptomic alterations in the DG of OVX rats. Without E2 replacement, SE altered expression of 44% of the DG genes. SE affected all major functional pathways, including apoptosis (61%), Alzheimer's disease (47%), cell cycle (59%), long-term potentiation (62%), and depression (55%), as well as synaptic vesicle cycle (62%), glutamatergic (53%), GABAergic (49%), cholinergic (52%), dopaminergic (55%), and serotonergic (49%) neurotransmission. However, in rats with E2 replacement the percentage of significantly affected genes after SE was reduced to the average 11% (from 8% for apoptosis to 32% for GABAergic synapse). Interestingly, while SE down-regulated most of the synaptic receptor genes in oil-injected females it had little effect on these receptors after E2-replacement. Our novel Pathway Protection analysis indicated that the E2-replacement prevented SE-related damage from 50% for GABA to 75% for dopaminergic transmission. The 15% synergistic expression between genes involved in estrogen signaling (ESG) and neurotransmission explains why low E2 levels result in down-regulation of neurotransmission. Interestingly, in animals with E2-replacement, SE switched 131 synergistically expressed ESG-neurotransmission gene pairs into antagonistically expressed gene pairs. Thus, the ESG pathway acts like a buffer against SE-induced alteration of neurotransmission that may contribute to the E2-mediated maintenance of brain function after the SE injury in postmenopausal women. We also show that the long-term potentiation is lost in OVX rats following SE but not in those with E2 replacement. The electrophysiological findings in OVX female rats with SE are corroborated by the high percentage of long-term potentiation regulated genes (62%) in oil-injected while only 13% of genes were regulated following SE in E2-replaced rats.

## Introduction

Special considerations are necessary for women with epilepsy. Irregularities in ovarian function (reflected in menstrual cycles), may affect the likelihood of seizures (Harden et al., 1999; Herzog et al., 2004; Velíšková and DeSantis, 2013). Especially women with seizures originating in temporal lobe (temporal lobe epilepsy, TLE) often have premature ovarian failure/early menopause (late thirties-early forties), which may affect seizure rate (Klein et al., 2001; Isojarvi, 2003; Pennell, 2009) and further contribute to cognitive decline, dementia, and vulnerability to neuronal dysfunction (Shuster et al., 2010; Rocca et al., 2011). The female sex hormones (especially β-estradiol, E2) have neuroprotective effects including attenuation of seizure-induced hippocampal damage and cognitive decline. The E2 effects involve complex transcriptomic mechanisms that are still poorly understood. Therefore, estrogen effects on seizures and their consequences need to be further investigated to determine beneficial effects of hormone therapy in women with epilepsy.

In this report, we analyze alterations in neurotransmission transcriptome of the hippocampal dentate gyrus (DG) induced by status epilepticus (SE) in ovariectomized (OVX) female rats and compare to changes in OVX rats with E2 replacement. In addition to the standard analyses based on our genomic fabric paradigm approach (Iacobas et al., 2012, 2017; Iacobas, 2016), we used also novel quantifiers termed Weighted Pathway Regulation (WPR) and Pathway Protection (PPR). The new quantifiers are not affected by the arbitrarily introduced cut-offs for the absolute fold-change and *p*-value to consider a gene as significantly regulated. We focused on the interplay between the estrogen signaling (ES) pathway and five types of neurotransmission: glutamatergic (GLU), GABAergic (GABA), dopaminergic (DA), cholinergic (ACH), and serotonergic (5HT). Since estrogen acts through estrogen receptors, which are ligand-induced transcription factors, we wanted to determine the extent of its effects on gene transcription under condition of SE-induced hippocampal damage.

In order to understand the magnitude of the SE-related damage and the protection provided by the E2 we have included also data on several other pathways: apoptosis (APO), cell cycle (CC), synaptic vesicle cycle (SVC), long-term potentiation (LTP), long-term depression (LTD), and Alzheimer's disease (ALZ).

Because estrogen signaling ESG and neurotransmission have several genes in common, we considered these common genes as the main vehicles through which ESG regulates the synaptic connection and as such, the brain circuitry. The common genes were identified using the Kyoto Encyclopedia of Genes and Genomes developed by Kanehisa Laboratories (Kanehisa et al., 2017). These genes are: adenylcyclases (*Adcy2, Adcy3, Adcy5, Adcy6, Adcy7, Adcy8, Adcy9*), oncogenes (*Akt1, Ak3, Fos, Hras, Kras, raf1, Src*), transcription factors (*Atf4, Atf6b*), calmodulins (*Calm1, Calm2, Calm3*), membrane receptors (*Gabbr1, Gabbr2, Grm1, Itpr1, Itpr2*), binding proteins (*Creb1, Creb3l1, Gnai1, Gnai2, Gnai3, Gnao1, Gnaq*), ion channels (*Kcnj3, Kcnj5, Kcnj9*), and kinases (*Map2k1, Mapk1, Mapk3, Pik3ca, Pik3cb*).

In addition, we show the complex transcriptomic regulation of the hippocampal DG neurotransmission by E2 replacement that is functionally important to preserve neuronal plasticity following SE. Results bring essential insights into E2-neurotransmission interactions, which are interesting beyond the seizures for understanding sex-specific and hormonal modulated depression/anxiety, stress processing, memory, and cognition.

## Materials and methods

### Animals

Experiments were carried out according to the Revised Guide for the Care and Use of Laboratory Animals and approved by the New York Medical College Animal Care and Use Committee. We used 8–9 week old female Sprague-Dawley rats (Taconic Farms) kept on a 12-h light/dark cycle (lights on at 07:00 a.m.) with food and water *ad libitum*. Rats were OVX (= castrated) under ketamine/xylazine (70/10 mg/kg intraperitoneally) anesthesia and, 1 week later, injected subcutaneously with either 17β-estradiol benzoate (2 μg/0.1 ml/day; Sigma-Aldrich, St. Louis, MO) or sterile peanut oil (0.1 ml; controls) daily at 10:00 for 4 consecutive days (Velíšková and Velíšek, 2007), a treatment producing E2 levels within a physiological range (Neal-Perry et al., 2005). Measurements of vaginal impedance confirmed the successful OVX as well as E2-replacement.

**Figure 1:**
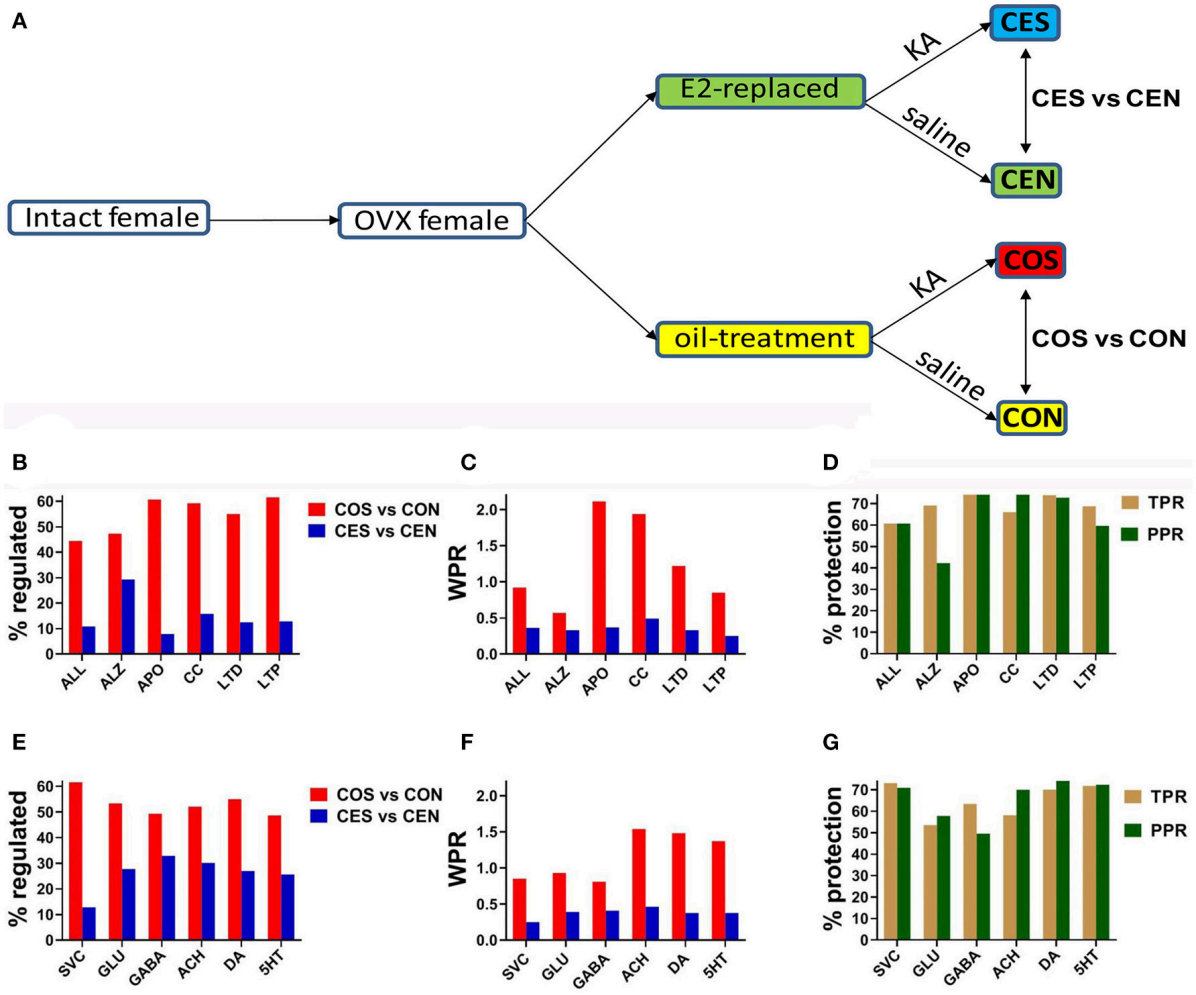
**(A)** Experimental design. OVX, ovariectomy; KA, kainic acid; saline, KA vehicle. Animals were injected with oil or 2 μg of β-estradiol benzoate for 4 days subcutaneously. **(B,E)** Percentage of the regulated genes within the entire transcriptome (ALL) and included in the analyzed pathways in CES and COS groups with respect to CEN, respectively CON groups. ALZ, Alzheimer's disease, APO, apoptosis, CC, cell cycle, LTD, long-term depression, LTP, long-term potentiation, SVC, synaptic vesicle cycle, GLU, glutamatergic synapse, GABA, GABAergic synapse, ACH, cholinergic synapse, DA, dopaminergic synapse, 5HT, serotonergic synapse. Note the massive effect of SE on the gene expression. **(C,F)** WPR scores for selected pathways. In **(B,C,E,F)** lower is better (less damaging). **(D,G)** Protection against transcriptomic regulation in E2-replaced animals quantified by both TPR (percent reduction of the number of significantly regulated genes) and PPR (percent reduction of WPR). Note that DA had the largest (better) protection as quantified by PPR.

SE was induced by injection of kainic acid (12.5 mg/kg intraperitoneally) 24 h following the last oil/E2 injection. Animals were observed for seizure occurrence and only animals with at least2 h of continuous forelimb clonus seizures were included for further experiments. There were no differences in seizure severity or duration between the treatment groups as described previously (Velíšková and Velíšek, 2007). SE was terminated by diazepam (10 mg/kg i.p.) injection. Control animals received saline as well as diazepam injections. Tissue from all groups was collected 24 h after SE. As illustrated in Figure 1A, studies were performed on four groups of OVX female rats: CON (castrated, oil-injected, no SE), CEN (castrated, E2-replaced, no SE), COS (castrated, oil-injected, SE), and CES (castrated, E2-replaced, SE). CON and CEN animals were obtained as previously described (Velíšková et al., 2015). All rats were decapitated under light isoflurane anesthesia. Separate groups of identically treated female rats were used for *in vitro* electrophysiology experiments.

### Electrophysiology *in vitro*

After decapitation, the brains were removed. Transverse combined entorhinal cortex-hippocampal slices (400 μm thick) were cut with a vibratome (Leica model DTK-1000) in ice-cold artificial cerebrospinal fluid (aCSF; in mM: NaCl 126, KCl 5, NaH_2_PO_4_ 1.25, MgCl_2_ 2, CaCl_2_ 2, NaHCO_3_ 26, and glucose 10; pH 7.36). Recordings started following 1 h pre-incubation in an interface chamber in a pre-warmed (34–35°C) and oxygenated (5% CO_2_/95% O_2_) aCSF. Field excitatory postsynaptic potentials (fEPSPs) were recorded with a glass microelectrode (2 M NaCl; 2–5 MΩ) placed in the middle third of the DG molecular layer as described previously (Nebieridze et al., 2012). Medial perforant path axons were stimulated once each 30 s with a bipolar stainless-steel stimulating electrode (FHC Inc.; Bowdoinham, ME). Baseline fEPSPs were recorded using a stimulus intensity that produced ~30% of maximal fEPSP slope. LTP was induced by theta burst stimulation (TBS; 10 bursts delivered at interburst interval 200 ms; each burst consisted of 10 stimuli at 100 Hz). The stimulus intensity has not been changed during the TBS. Picrotoxin (50 μM; Abcam, Cambridge, MA, USA) was bath applied during the experiment to enhance the LTP.

### Microarray

Two hours after the onset of continuous seizures, under light isoflurane anesthesia the rats were decapitated, brains were removed and hippocampal DG were isolated. Four animals were used from each of the four groups of OVX female rats (CON, CEN, COS, and CES). Previously described experimental protocol (Kravchick et al., 2016) was used for RNA extraction, purification, fluorescent labeling and reverse transcription, and hybridization with Agilent Rat GE 4 × 44k v3 microarrays in the “multiple yellow” design (Iacobas et al., 2006). In this design, differently labeled biological replicates are co-hybridized with each microarray. A non-control microarray spot is valid if the foreground fluorescence is at least twice its background. We normalize the background-subtracted fluorescence of each valid spot to the median of all same-label valid spots in the array, and compute the average for all spots probing the same transcript (thus eliminating the array redundancy). Same label average values for each transcript are then compared across experimental conditions and results averaged for the two colors. An in-house developed iterative algorithm renormalizes all data sets across the fluorescent labels and experimental conditions until the average change of expression ratios between successive iterations falls below 5%.

### Functional pathways

We used Kyoto Encyclopedia of Genes and Genomes (KEGG) maps developed by Kanehisa Laboratories (Kanehisa et al., 2017) to select the genes responsible for estrogen signaling pathway (map04915) and for cholinergic (map04725), glutamatergic (map04724), GABAergic (map04727), dopaminergic (map04728), and serotonergic (map04726) transmissions. KEGG maps were also used for apoptosis (map04210), cell cycle (map04110), synaptic vesicle cycle (map04721), long-term potentiation (map04720), long-term depression (map04730), and Alzheimer's disease (map05010) pathways. Although KEGG designed map04730 for the cerebellar long-term depression the basic molecular mechanisms are likely to occur also in the DG.

### Data analysis

According to our laboratory standard (Lee et al., 2017), a gene was considered as regulated (here by SE in oil-injected/E2-replaced females with respect to the corresponding non-SE animals) if the absolute fold-change (FC) exceeded the combined contributions of the technical noise and biological variability (CUT, cut-off). The regulation was considered significant if the *p*-value of the heteroscedastic *t*-test of the equality of the average expression levels in the compared conditions with the Bonferroni-type correction for redundant spots probing the same gene (Iacobas et al., 2005) was below 0.05. This FC cut-off computed for every single gene includes in the regulated list the “false negatives” of the traditionally uniform 1.5 × (very stably expressed genes across biological replicates that are probed by very clean spots with 1.5 > FC > CUT). In addition, it eliminates most of the “false positives” from the variably expressed genes that are also probed by not so clean spots (1.5 < FC < CUT).

In addition to the percentages of up-/down-regulated genes, we have also computed the Weighted Pathway Regulation (WPR) and Pathway Protection (PPR, similar to Pathway Restoration Efficiency) that we have recently introduced (Iacobas et al., 2017). WPR and PPR are free of the arbitrary FC and *p*-value cut-offs to consider a gene as significantly regulated and take also into account the magnitude and significance of each gene regulation. Thus, a gene with 2 × fold-change at *p*-val = 0.01 contributes to WPR more than twice than a gene with 1.5 × fold-change at *p*-val = 0.05. This approach is different from the percentage of regulated genes that not only depends on the arbitrarily introduced cut-offs but treats all regulated genes as equal contributors. We have used also the Transcriptomic Protection (TPR), similar to the Transcriptomic Recovery Efficacy (Lachtermacher et al., 2011; Soares et al., 2011), representing the reduction of the percentage of regulated genes while considering also the side effects (i.e., the percentage of the regulated genes by the E2-replacement alone).

### Transcriptomic networks

For each condition (CON, COS, CEN, CES) we determined how much expression of each gene is coordinated with expression of each other gene by computing Pearson correlation coefficient between the expression levels of the two genes among biological replicates. Expression coordination of two genes may result from several molecular mechanisms that may include (but it is not restricted to) the regulation of the expressions of both genes by a common transcription factor. We used coordination analysis (Iacobas et al., 2007a; Iacobas and Iacobas, 2010) to determine the transcriptomic networks by which genes in the estrogen signaling pathway controls the genes involved in all types of neurotransmission in the female DG. The analysis is based on our principle of “transcriptomic stoichiometry” (Iacobas et al., 2007b; Iacobas, 2016) stating that genes whose encoded proteins are working together in functional pathways should be coordinately expressed to produce the pathway players in the right proportions. In a previous paper (Iacobas et al., 2007c), we have shown that the transcriptomic networks do not stop at the cellular border. Instead, they may cross it to link pathways from (phenotypically similar or distinct) neighboring cells via intercellular signaling (Iacobas et al., 2006d).

## Results

We quantified expressions of 12,710 distinct genes in all 16 samples and computed 80,765,695 Pearson correlation coefficients among these genes for each of the four conditions. Detailed experimental procedure and raw and normalized gene expression data are publically available at http://www.ncbi.nlm.nih.gov/geo as GSE60013 and GSE107725.

### Gene expression regulation by SE and protection by E2 replacement

Figures 1B,E present the percentage of the SE-related regulated genes from all analyzed pathways in COS and CES groups with respect to their corresponding controls (CON and CEN groups). Of note is the substantial reduction of the percentage of regulated genes in OVX rats with EB replacement compared to the animals that received only oil. Note that larger percentages of genes were altered synaptic pathways than the overall alteration percentage in the entire transcriptome. Figures 1C,F present the WPR scores for selected pathways, confirming the substantially lower alteration of these pathways in rats with E2 replacement. Figures 1D,G present the protection against SE alteration in E2 replaced animals as quantified by both TPR and PPR.

**Figure 2:**
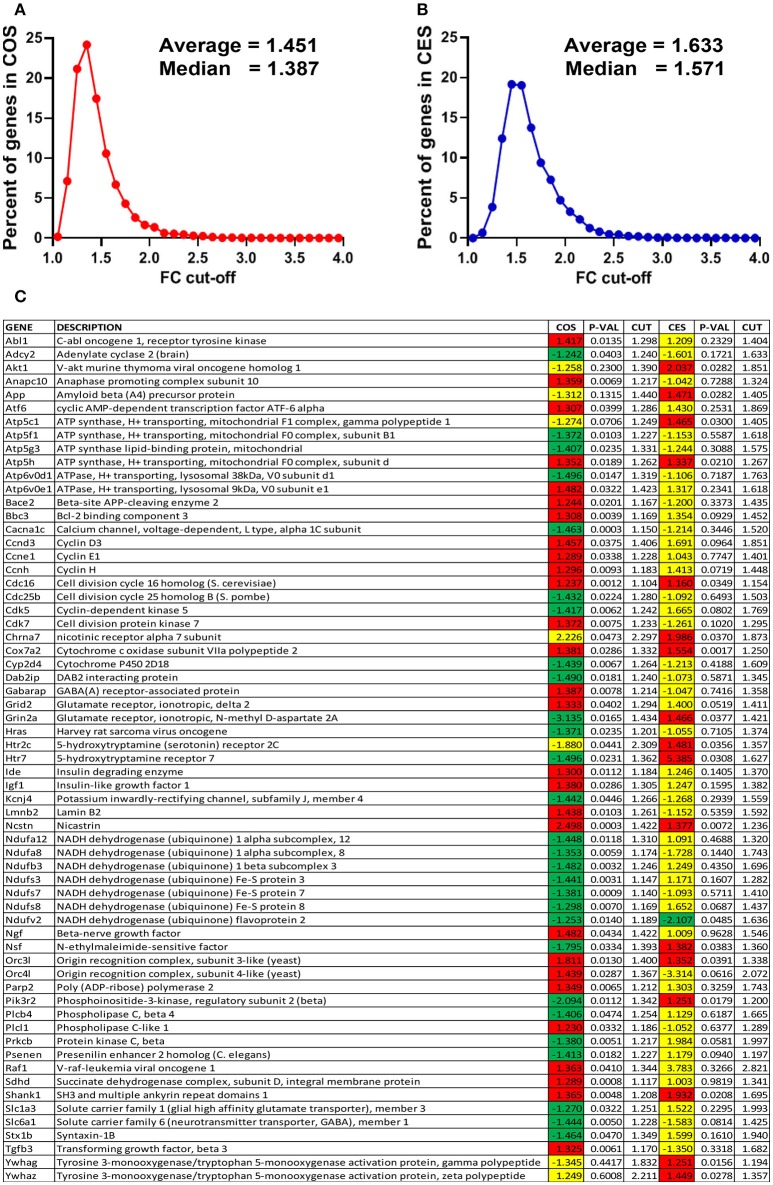
Power of the flexible fold-change cut-off. **(A)** Distribution of the fold-change cut-offs for the COS vs. CON comparison. COS, expression ratio in the comparison COS vs. CON, CES, expression ratio in the comparison CES vs. CEN. When all 12,710 quantified unigenes are considered, CUT values range from 1.055 for *Pcyt1a* to 4.062 for *Scgb1c1*, with the average 1.451. **(B)** Distribution of the fold-change cut-offs for the CES vs. CEN comparison. When all 12,710 quantified unigenes are considered, CUT values range from 1.109 for *Mapk1ip1l* to 3.877 for *Ttr*, with the average 1.633. **(C)** “False negatives” and “false positive” regulation hits from the analyzed functional pathways. Note that expression ratio 2.226 was not enough for *Chrna7* to be considered as up-regulated in COS vs. CON comparison (CUT = 2.297) but 1.986 was enough in CES vs. CEN (CUT = 1.873).

The expression ratios (x, negative for down-regulation), the absolute fold-change cut-offs (CUT) and the *p*-values for all regulated genes in the analyzed pathways are listed in the Supplementary Table S1. In order to illustrate how our method of flexible FC cut-off works, we present in Figure 2 the distributions of the FC cut-offs and the list of “false negatives” (e.g., *Abl1* with 1.5 > COS = 1.477 > CUT = 1.298) and “false positives” (e.g., *Chrna7* with 1.5 < COS = 2.226 < CUT = 2.297) selected from the Supplementary Table S1. Of note from Figure 1C is that the FC ranges from 1.104 (for the “false negative” *Cdc16* in COS) to 2.821 (for *Raf1* in CES).

In addition to regulate the expression of thousands genes, both SE and E2 replacement separately or together turned ON/OFF many other genes. Table 1 lists the turned on/off genes by E2 replacement alone in non-SE rats and the genes whose turning on by SE was prevented by E2 replacement.

**Table 1:** Genes turned on/off by E2 replacement alone (in non-SE rats) and genes turned on by SE (in COS animals) but prevented by E2 replacement (CES rats).

**Gene**	**Symbol**
**Genes turned on by estradiol replacement alone**	
Chemokine (C-C motif) receptor 10	Ccr10
Fibronectin type III domain containing 5	Fndc5
G protein-coupled receptor 39	Gpr39
Short coiled-coil protein	Scoc
Unc-5 homolog C (*C. elegans*)	Unc5c
**Genes turned off by estradiol replacement alone**	
Leukocyte cell derived chemotaxin 1	Lect1
**Genes whose turned on by se was prevented by estradiol replacement**	
ARP1 actin-related protein 1 homolog B (yeast)	Actr1b
A disintegrin and metalloprotease domain 4	Adam4
ArfGAP with GTPase domain, ankyrin repeat, and PH domain 1	Agap1
Adenosylhomocysteinase-like 1	Ahcyl1
Aldehyde dehydrogenase family 1, subfamily A7	Aldh1a7
Amyotrophic lateral sclerosis 2 (juvenile) homolog (human)	Als2
ATPase, H+ transporting, lysosomal V0 subunit A2	Atp6v0a2
Dopamine receptor D2	Drd2
Family with sequence similarity 53, member B	Fam53b
Forkhead box C2	Foxc2
Gonadotropin-releasing hormone 1 (luteinizing-releasing hormone)	Gnrh1
HECT, C2 and WW domain containing E3 ubiquitin protein ligase 2	Hecw2
Helicase, lymphoid specific	Hells
Hepatocyte growth factor	Hgf
Minichromosome maintenance complex component 4	Mcm4
Myelin gene regulatory factor	Mrf
Myosin, heavy chain 10, non-muscle	Myh10
Nuclear transcription factor-Y alpha	Nfya
Patatin-like phospholipase domain containing 3	Pnpla3
Surfactant protein C	Sftpc
Sarcoglycan zeta	Sgcz
Seven in absentia 1A	Siah1a
SLAM family member 8	Slamf8
Solute carrier family 39 (zinc transporter), member 14	Slc39a14
Syntaxin 2	Stx2
SMT3 suppressor of mif two 3 homolog 3 (*S. cerevisiae*)	Sumo3
Transcription factor AP-2 beta	Tcfap2b
Tetratricopeptide repeat domain 33	Ttc33
Ubiquitin-associated protein 2	Ubap2
Unc-5 homolog D (*C. elegans*)	Unc5d
Zinc finger, C4H2 domain containing	Zc4h2
Zinc metallopeptidase, STE24 homolog (*S. cerevisiae*)	Zmpste24
Zinc finger, MYM-type 4	Zmym4
Zinc finger protein 286A	Znf286a

### Effects of SE on the LTP in the hippocampal DG of OVX oil-injected and E2 replaced female rats

**Figure 3:**
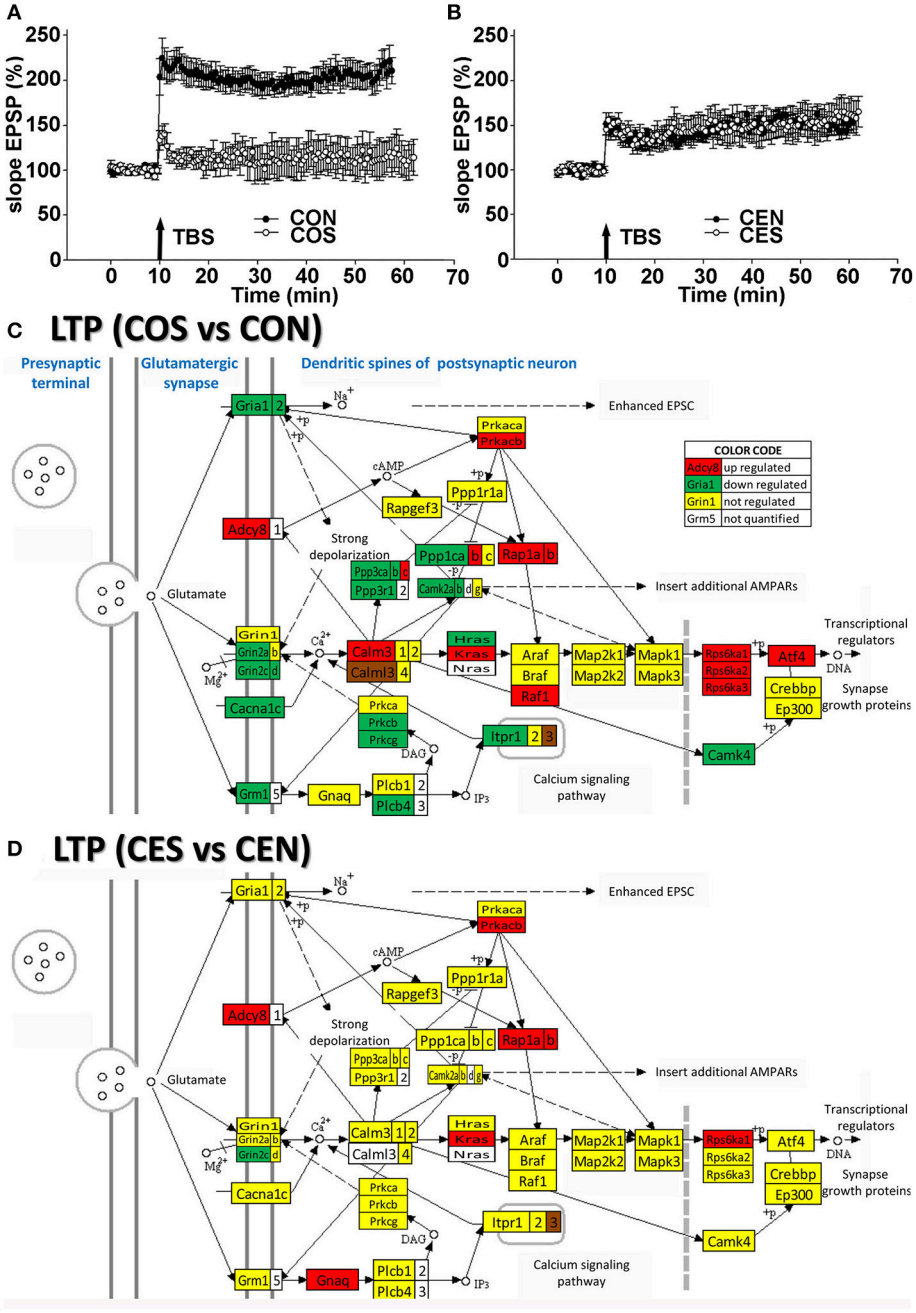
Electrophysiology and transcriptomics of the long-term potentiation (LTP). **(A,B)** Medial perforant path LTP in the DG in OVX rats is lost following SE but preserved in E2-replaced animals. LTP induced by TBS following SE (filled circles) compared to rats with no seizures (open circles). An arrow marks a moment when the TBS was administered. In slices from oil-injected rats exposed to SE (COS; *n* = 5), TBS-induced LTP was severely reduced compared to LTP from rats with no SE (CON; *n* = 10). In contrast, the magnitude of TBS-induced LTP in slices from E2-replaced OVX rats following SE (CES; *n* = 5) was not different from LTP in slices from rats with no seizure experience (CEN; *n* = 10). **(C,D)** Dramatic regulation of LTP associated genes by SE in OVX rats compared to their non-SE counterparts (COS vs. CON) is in contrast to protection against the transcriptomic regulation of LTP associated genes in E2 replaced animals (CES vs. CEN). As specified in the “COLOR CODE” inset table, red/green/yellow background of the gene symbol indicates up-/down-/no regulation, while the white background indicates that expression of that gene was not quantified. Note the significantly lower number of regulated genes in rats with E2 replacement.

To confirm the functional protection provided by the E2 replacement against SE, we compared the TBS-induced LTP in the hippocampal DG of animals with no SE experience to LTP from animals following kainic acid-induced SE. In oil-injected OVX females (no E2 replacement), we found that the TBS-induced LTP was lost following SE (Figure 3A) while in OVX rats with E2 replacement, the magnitude of the TBS-induced LTP in the DG was not affected by SE (Figure 3B). These data are in accord with the transcriptomic findings (Figures 3C,D). Thus, the LTP data clearly demonstrate that the DG plasticity was functionally severely compromised and associated with dramatic gene regulation in the LTP pathway following SE in OVX animals. In contrast, the SE did not disturb the LTP in animals with E2 replacement.

### Regulation of neurotransmission pathways by SE w/o E2 replacement

**Figure 4:**
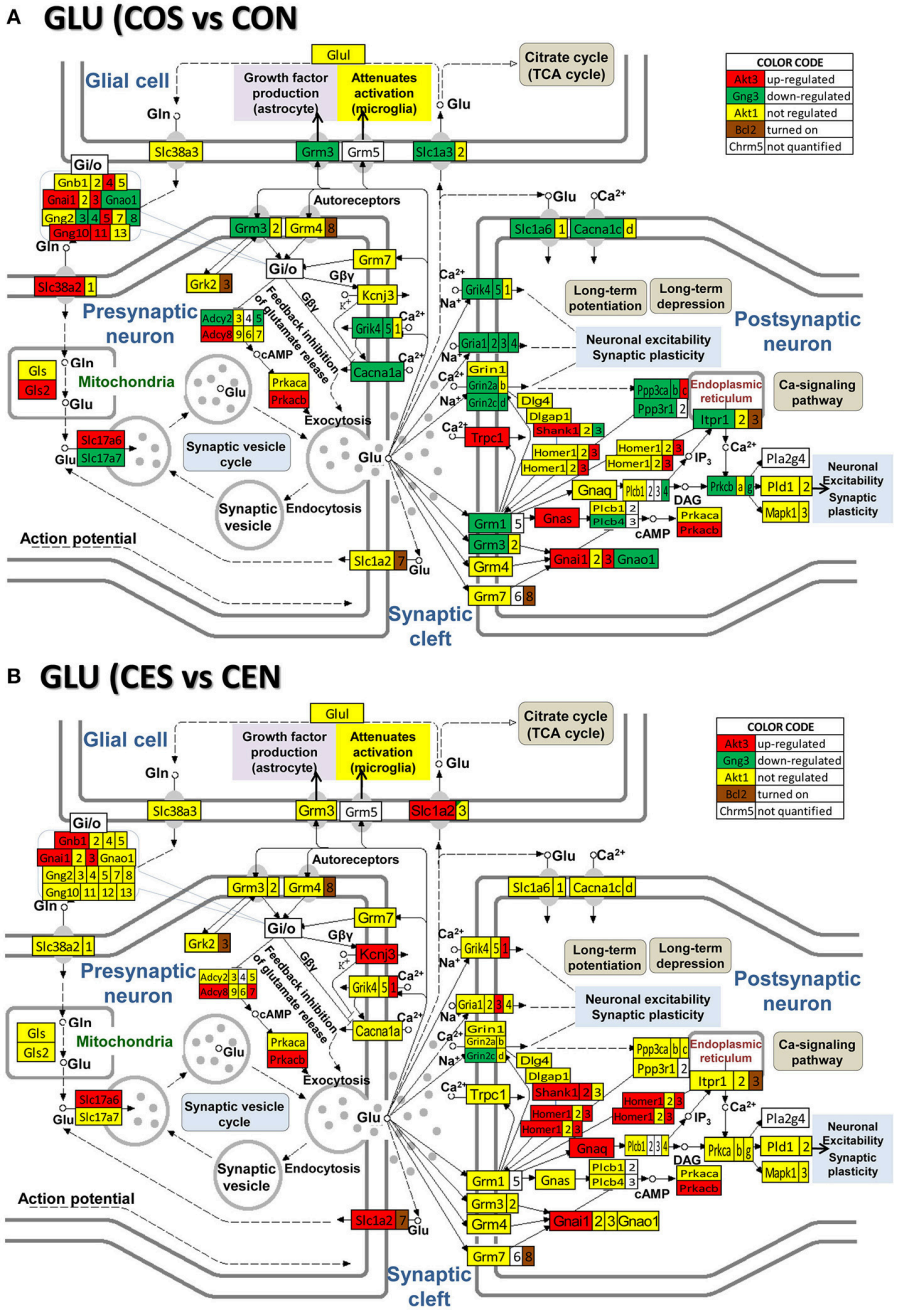
Regulation of the glutamatergic synapse pathway by SE in OVX rats with (CES)/without (COS) E2-replacement with respect to their non-SE counterparts (CEN and CON). Note the large number of down-regulated genes by SE in oil injected rats [e.g., metabotropic glutamate receptor 3, *Grm3* in **(A)**] whose expression was not affected by SE in rats with E2 replacement **(B)**.

**Figure 5:**
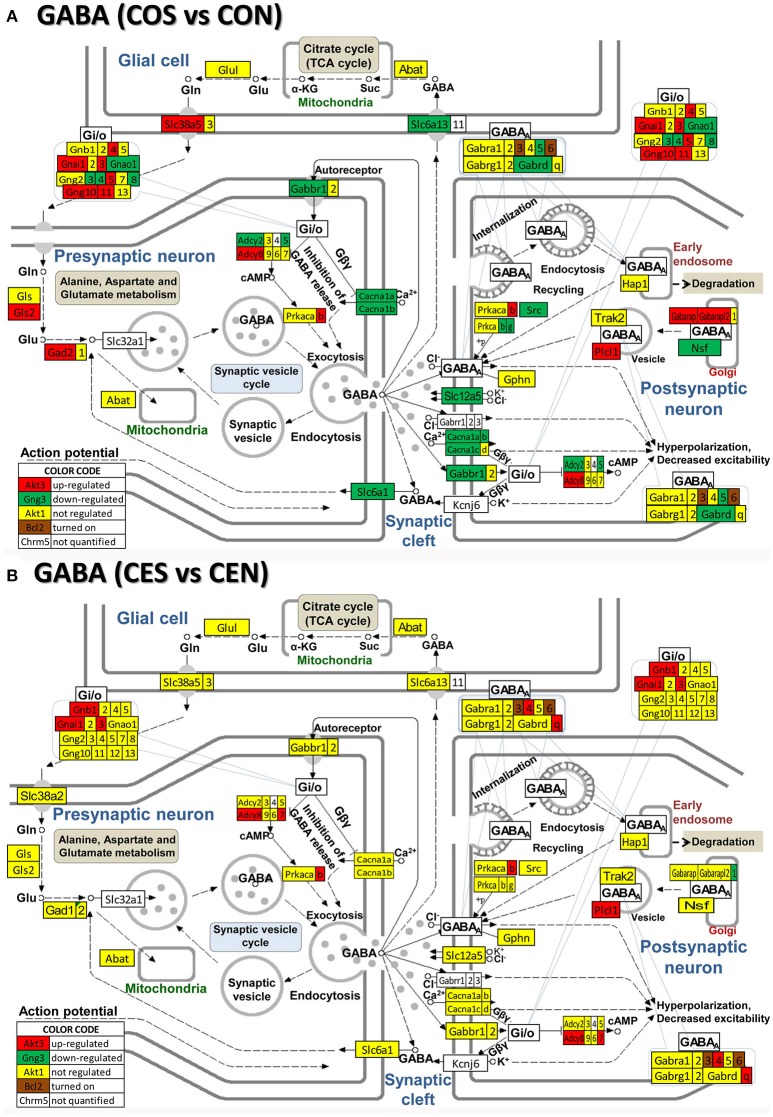
Regulation of the GABAergic synapse pathway by SE in OVX rats with (CES)/without (COS) E2-replacement with respect to their non-SE counterparts (CEN and CON). Note large number of genes down-regulated by SE in oil-injected animals [e.g., GABA B receptor 1, *Gabbr1* in **(A)**] whose expression was not affected by SE in rats with E2 replacement **(B)**.

**Figure 6:**
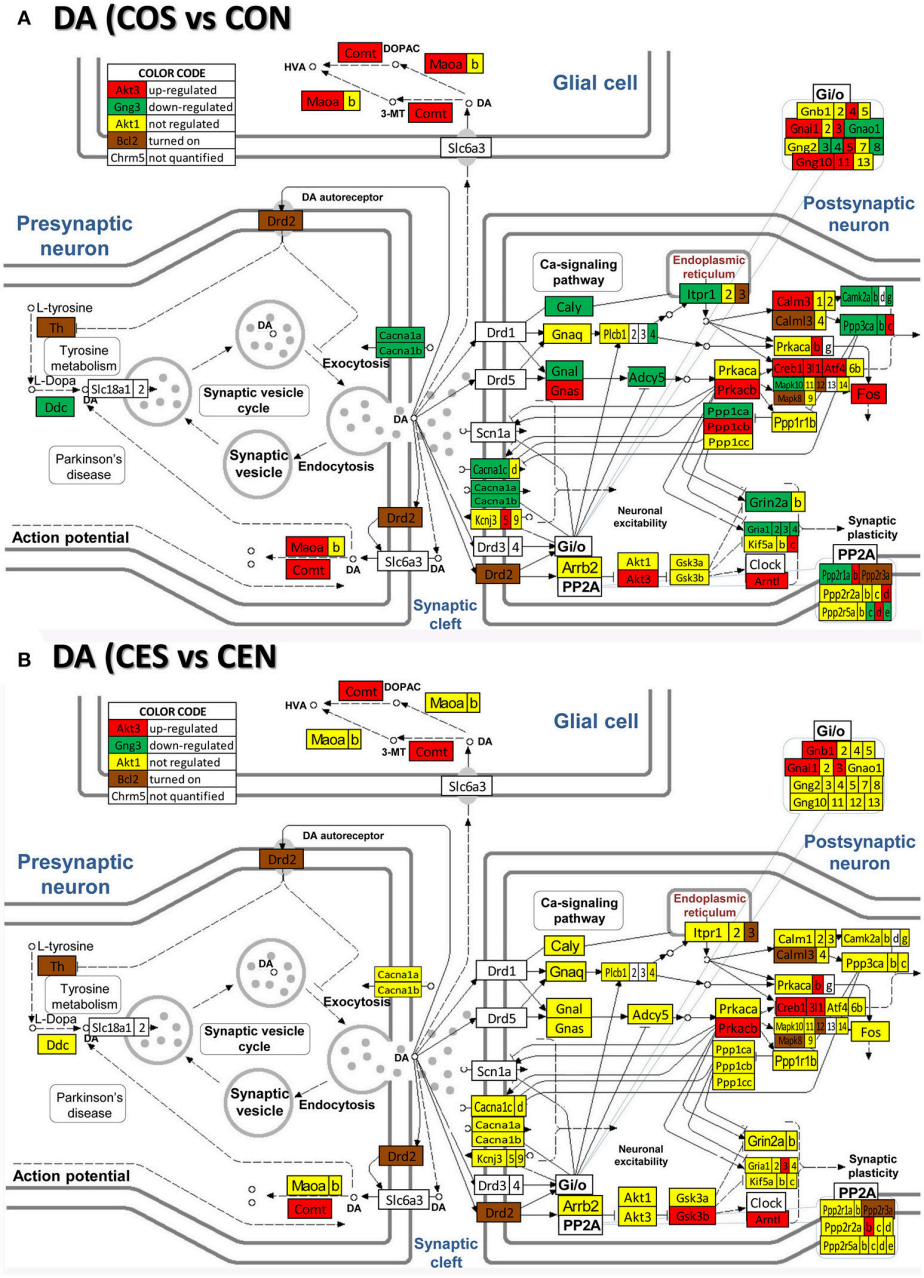
Regulation of the dopaminergic synapse pathway by SE in OVX rats with (CES)/without (COS) E2-replacement with respect to their non-SE counterparts (CEN and CON). Note the number of genes down-regulated by SE in oil-injected animals [e.g., DOPA decarboxylase, *Ddc* in **(A)**] whose expression was not affected by SE in rats with E2 replacement **(B)**.

**Figure 7:**
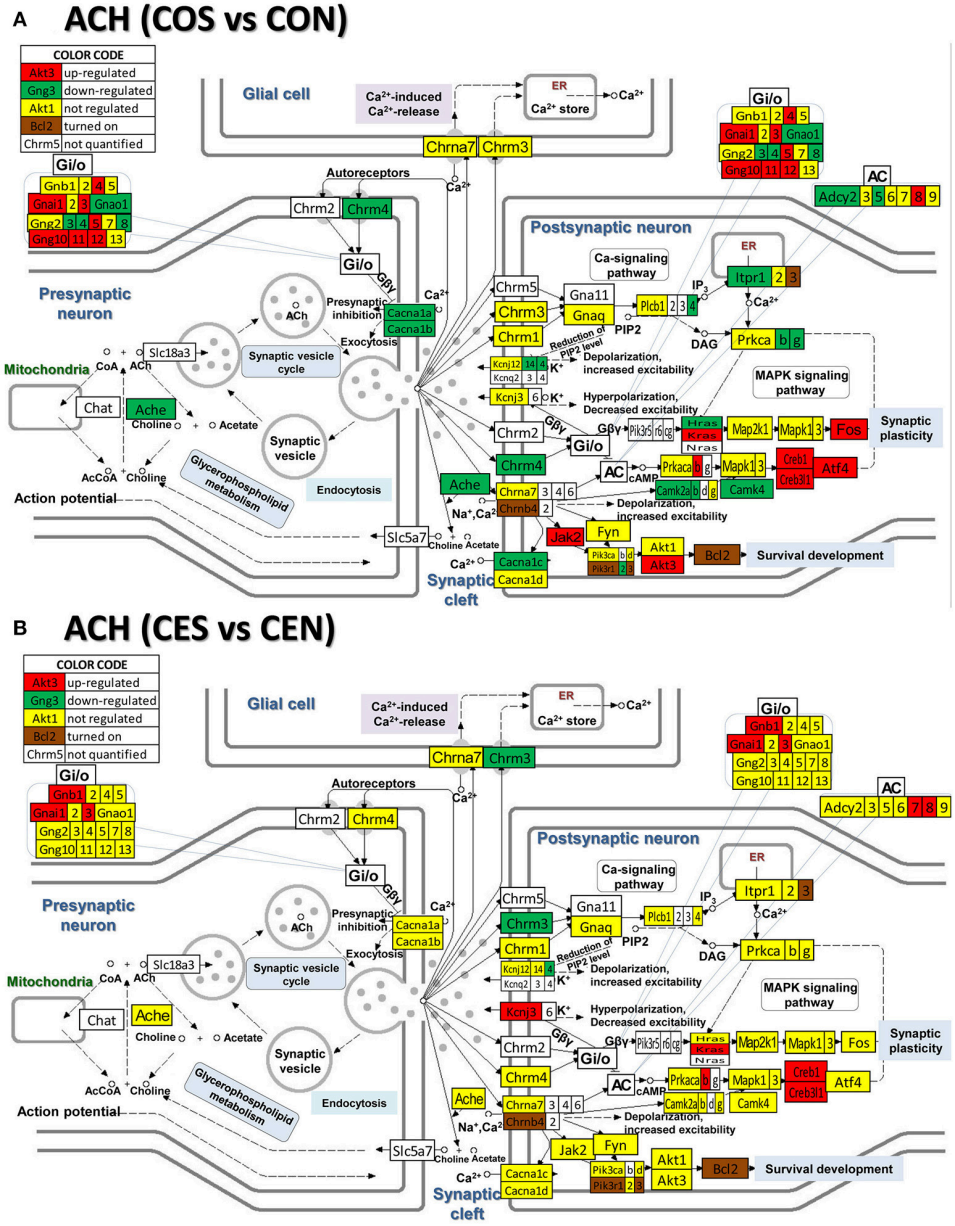
Regulation of the cholinergic synapse pathway by SE in OVX rats with (CES)/without (COS) E2-replacement with respect to their non-SE counterparts (CEN and CON). Again, note the large number of genes down-regulated by SE in oil-injected animals [e.g., acetylcholinesterase, *Ache* in **(A)**] whose expression was not affected by SE in rats with E2 replacement **(B)**.

**Figure 8:**
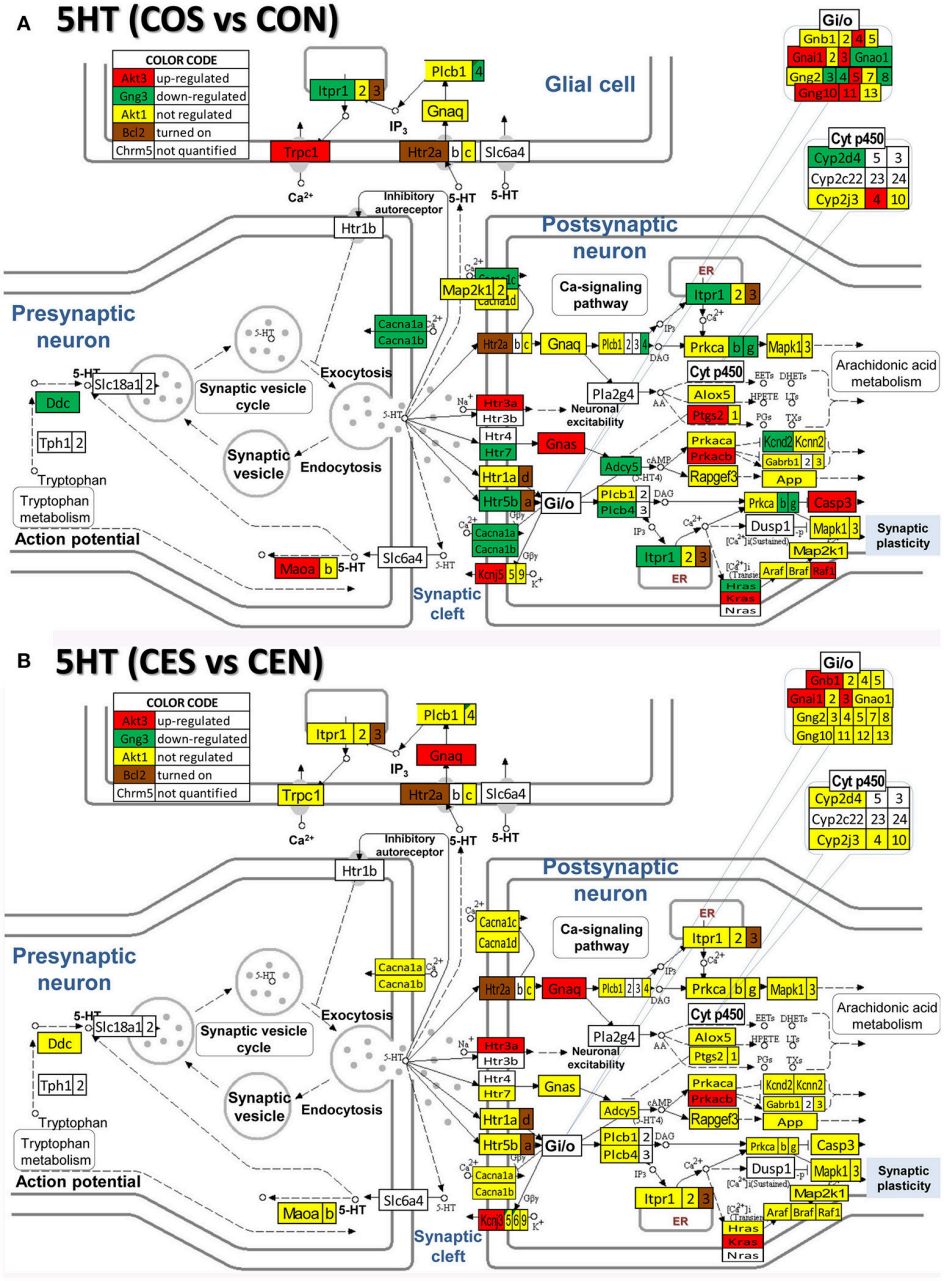
Regulation of the serotonergic synapse pathway by SE in OVX with (CES)/without (COS) E2-replacement with respect to their non-SE counterparts (CEN and CON). Note the number of down-regulated genes by SE in oil-injected animals [e.g. serotonin receptor 5B, *Htr5b* in **(A)**] whose expression was not affected by SE in rats with E2 replacement **(B)**.

Figures 4–8 present the regulation of GLU, GABA, DA, ACH, and 5HT pathways by SE in OVX rats w/o E2-replaced with respect to their non-SE counterparts. Of note is the large number (58!) of down-regulated genes in oil-injected animals whose expression was preserved in E2-replaced rats. The protected genes include: adenylate cyclases (*Adcy2, Adcy5*), calcium (*Cacna1a, Cacna1b, Cacna1c*), potassium (*Kcnd2, Kcnj14*), and solute carrier (*Slc12a5, Slc17a7, Slc1a3, Slc1a6, Slc6a1, Slc6a13*) channels and kinases (*Camk2a, Camk2b, Camk4, Mapk10, Pik33r2, Prkcb, Prkcg*). E2 replacement preserved also expression of several receptor genes: *Chrm4, Gabr1, Gabr5, Gabrd, Gnal, Gnao1, Gng3, Gng4, Gria1, Gria2, Gria4, Grik4, Grik5, Grin2a, Grin2d, Grm1, Grm3, Htr5b, Htr7, Itpr1*). Moreover, the down-regulation of *Gria3* in oil-treated animals was switched to up-regulation in E2-replaced. E2 replacement also protected some binding proteins (*Gnb4, Gng10, Gng11, Gng12, Gng5*) and oncogenes (*Akt3, Fos, Jun, Raf1*) against the SE-related upregulation. However, other genes, not affected by SE in oil-injected animals were regulated in E2-replaced ones (*Adcy7, Chrm3, Gabarapl1, Gabra4, Gabrq, Gnb1, Grik1, Gsk3b, Homer1, Kcnj3, Ppp2r2b, Shank2, Slc1a2, Sos2*). The protection against SE-related down-regulation of *Itpr1* and calcium channels is particularly important for the role these genes play in the intercellular calcium signaling (Iacobas et al., 2006d).

**Figure 9:**
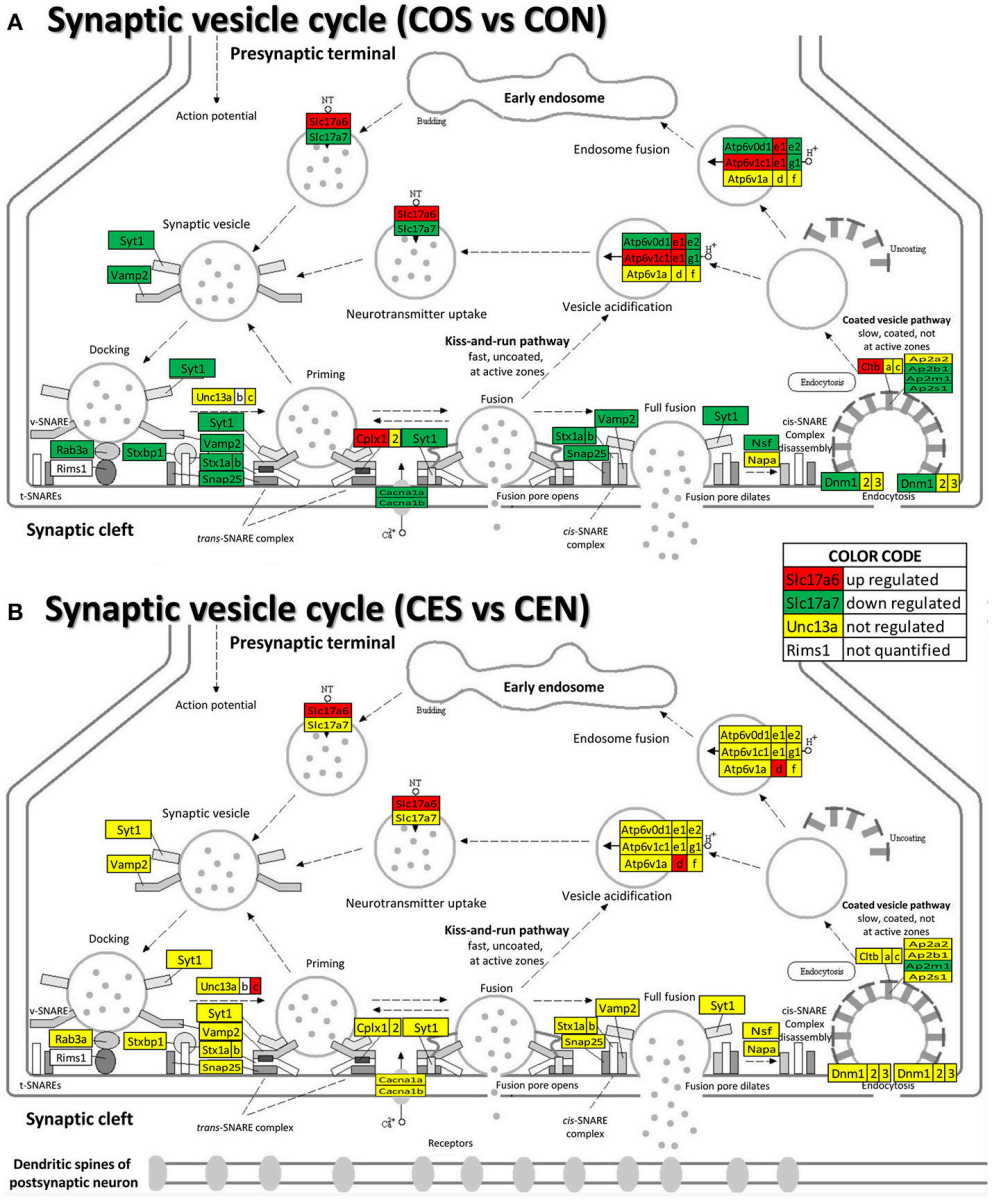
Regulation of the synaptic vesicle cycle pathway by SE in OVX with (CES)/without (COS) E2-replacement with respect to their non-SE counterparts (CEN and CON). Note that in oil-injected animals **(A)** the vesicle cycle was altered by SE in all phases but only few genes were affected in animals with E2 replacement **(B)**.

Our data show (Figure 9) that also the synaptic vesicle cycle (SVC) was massively altered by SE in oil-injected females and on much smaller scale in rats with E2 replacement.

### SE remodels the transcriptomic networks by which the ESG pathway controls neurotransmission

**Figure 10:**
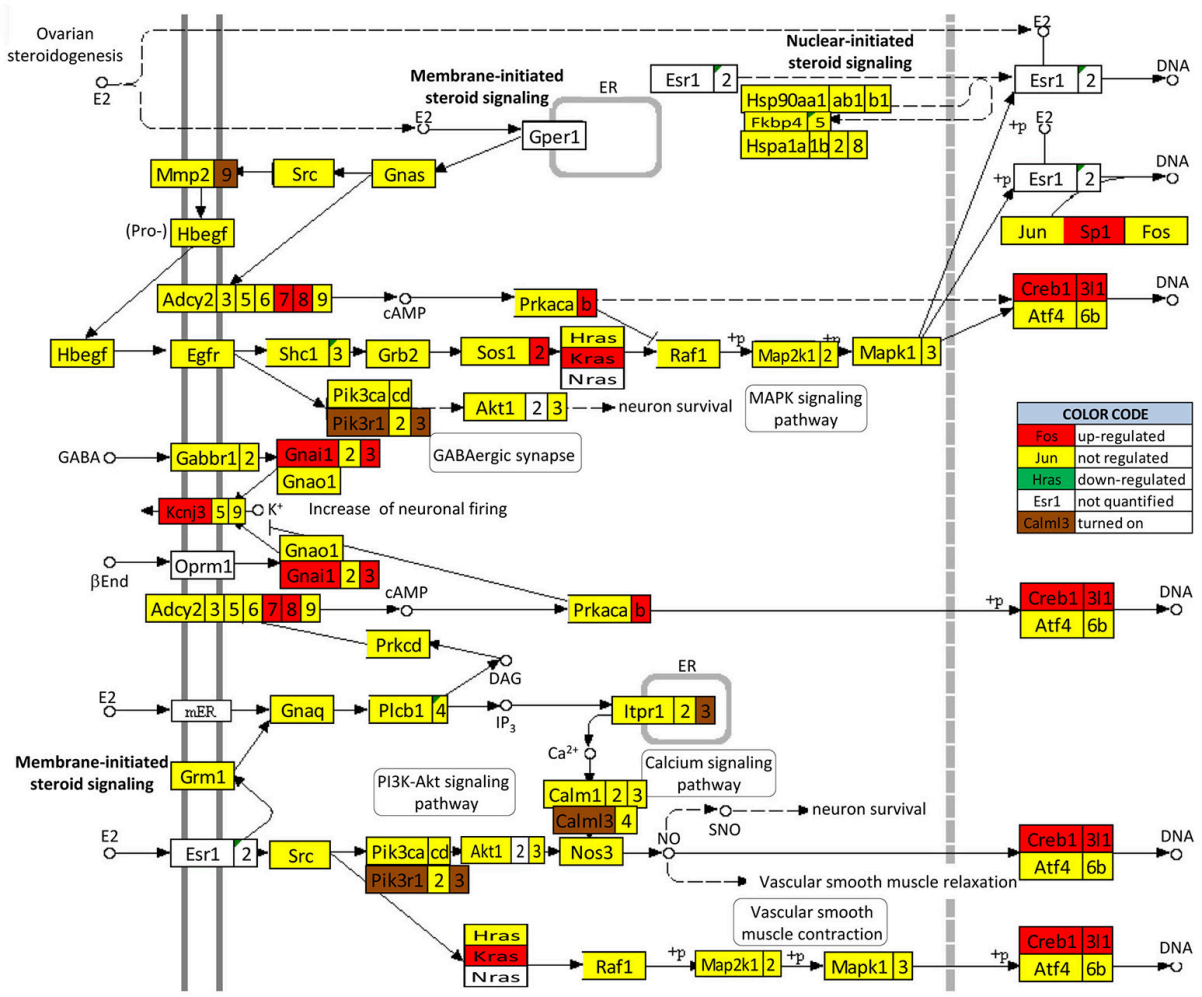
Regulation of the estrogen signaling pathway by SE in OVX females with E2 replacement. Note that, in addition to up-regulating *Adcy7, Adcy8, Creb1, Creb3l1, Gnai1, Gnai3, Kcnj3, Kras, Prkacb*, and *Sp1*, SE turned on *Calml3, Itpr3, Mmp9, Pik3r1*, and *Pik3r3*. No gene of this pathway was found as down-regulated or turned off by SE.

We found that SE not only regulates the ESG pathway (Figure 10) but also profoundly remodeled the transcriptomic networks by which ESG regulates neurotransmission. Interestingly, in animals with E2 replacement, SE turned on the very important *Pik3r1* and *Pik3r3* [phosphoinositide-3-kinase regulatory subunits 1 (alpha) and 3 (gamma)] that control numerous major pathways via the PI3K-AKT signaling.

**Figure 11:**
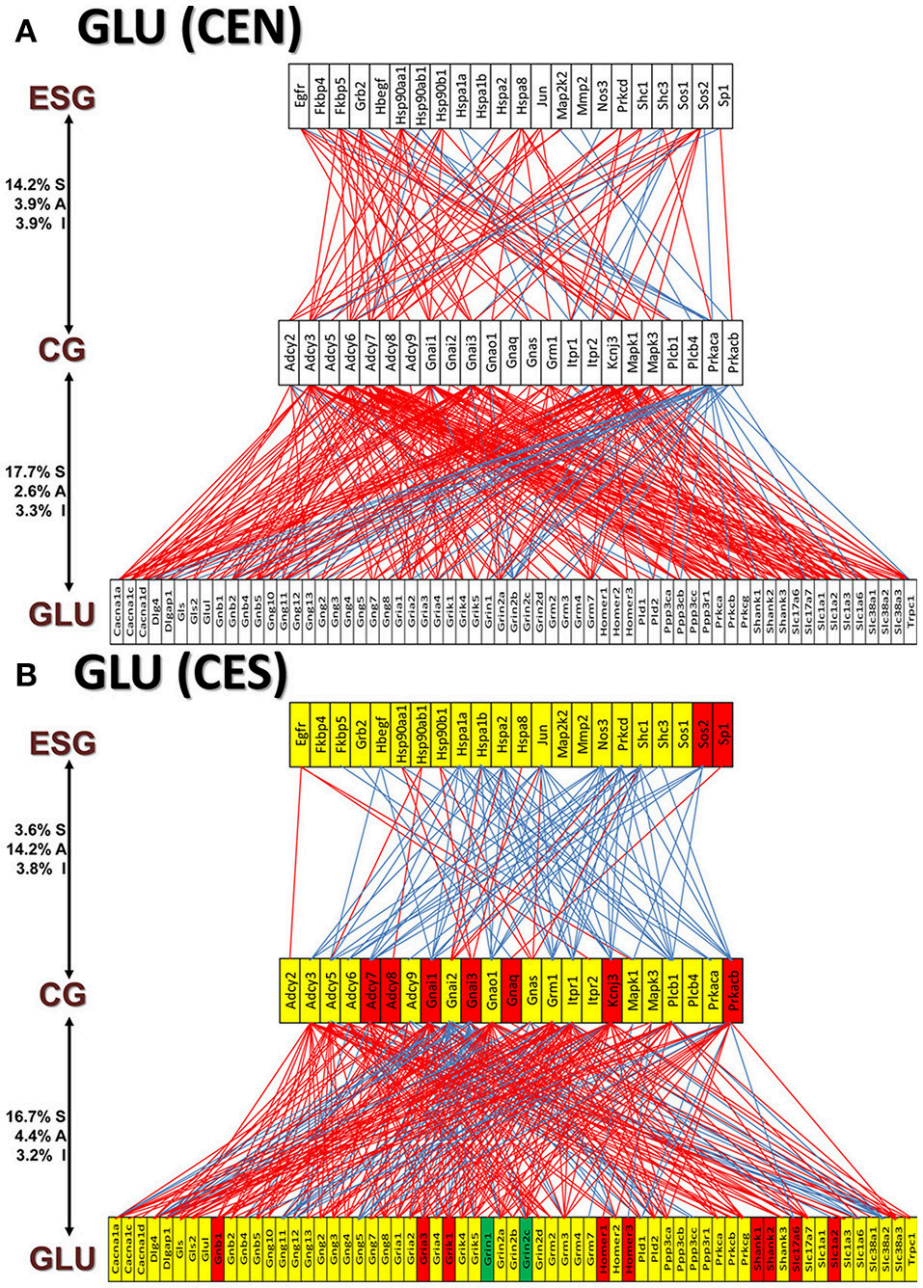
The transcriptomic networks by which the estrogen-signaling (ESG) pathway genes regulate the glutamatergic synapse (GLU) genes through expression coordination with the common genes (CG) between the two pathways in OVX rats with E2-replacement. **(A)** Networks in the DG of non-SE animals. A red/blue line indicates that the linked genes are significantly (*p*-value < 0.05) synergistically/antagonistically expressed, respectively. Numbers on the left side represent the percentages of the synergistically (S)/antagonistically (A)/independently (I) expressed pairs that can be formed between the two groups of genes. **(B)** Networks in the DG of SE animals. Red/green/yellow background of the gene symbol indicates that that gene was up-/down-/not regulated by SE. Note that there is a substantial shift from synergistic to antagonistic expression between ESG and CG groups in SE animals.

**Figure 12:**
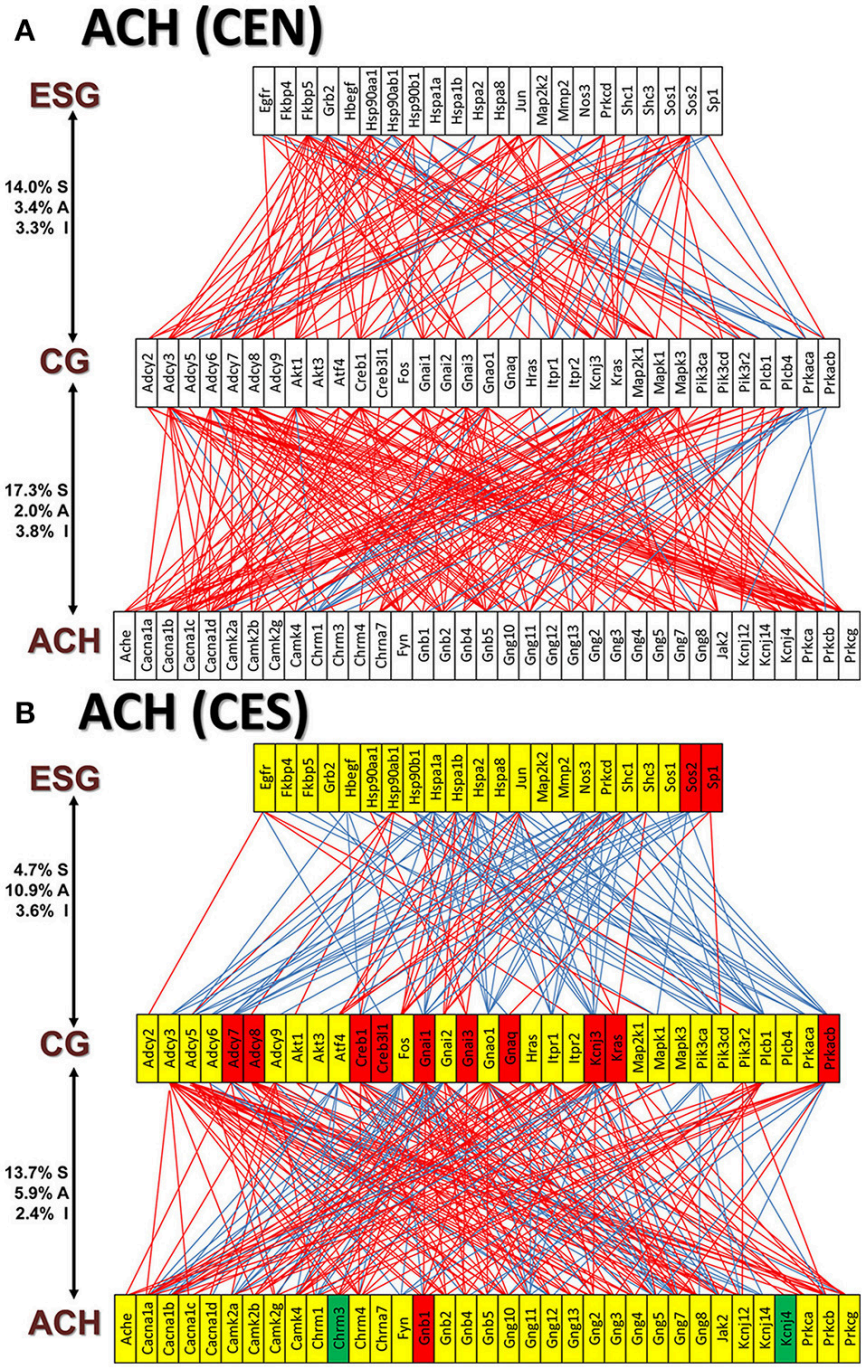
The transcriptomic networks by which the estrogen-signaling (ESG) pathway genes regulate the cholinergic synapse (ACH) genes through expression coordination with the common genes (CG) between the two pathways in OVX rats with E2-replacement. **(A)** Networks in the DG of non-SE animals. **(B)** Networks in the DG of SE animals. Note the substantial shift from synergistic to antagonistic expression between ESG and CG groups in SE animals.

Figures 11A and 12A present the transcriptomic networks that interlink ESG pathway genes with glutamatergic and cholinergic transmission via common genes in OVX animals with E2 replacement. Figures 11B, 12B show how SE altered these networks. The analysis revealed high degree (15%) of synergistic expression between genes involved in ESG pathway and all types of neurotransmission. This synergism means that when the ESG diminishes, the expression of neurotransmission genes decreases as well. This may explain why decline in E2 levels is associated with increased incidence of neurodegenerative diseases.

**Figure 13:**
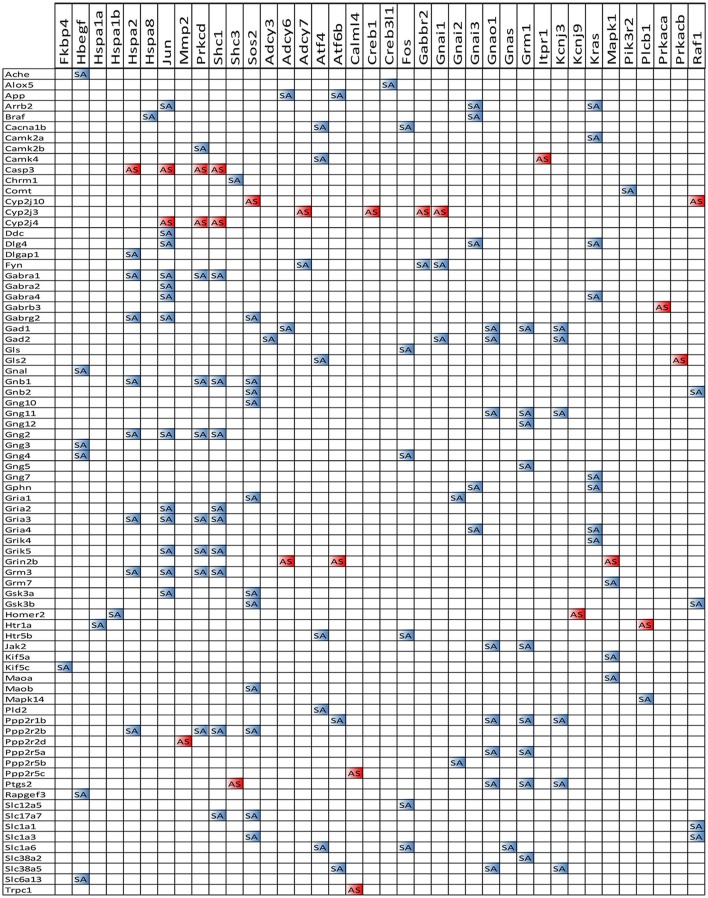
ES-neurotransmission gene pairs that switched their expression coordination from synergistic to antagonistic (white to blue bullets) or from antagonistic to synergistic (white to red bullets) following SE.

We also noticed that in animals with E2 replacement the synergistic expression of 131 ESG-neurotransmission gene pairs was switched by SE into antagonistic expression (albeit 25 pairs were switched from antagonistically to synergistically expressed). Thus, when expression of the ESG gene partner decreases, the expression of the paired neurotransmission gene goes up, likely compensating for the decrease of other synapse molecular factors. Figure 13 presents the ESG-neurotransmission gene pairs that switched their expression coordination from synergistic to antagonistic or *vice versa* in E2-replaced animals following SE. For instance, the transcription factor *Jun*, known for its role in synaptic plasticity and term memory formation (Alberini, 2009) had 13 synergistic-to-antagonistic switch(with: *Arrb2, Ddc, Dlg4, Gabra1, Gabra2, Gabr4, Gabrg2, Gng2, Gria2, Gria3, Grik5, Grm3, Gsk3a*) and 2 antagonistic-to synergistic ones (*Casp3, Cyp2j3*).

**Figure 14:**
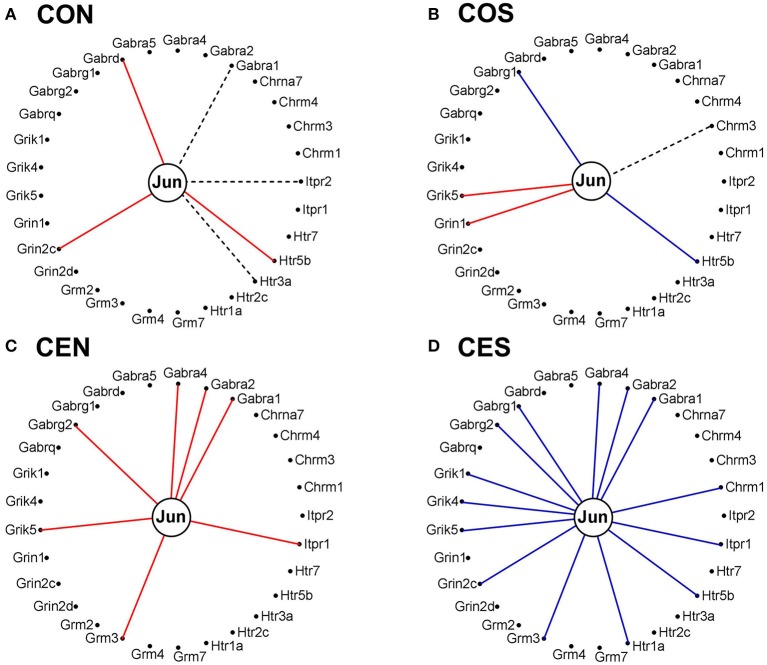
Expression correlation of Jun oncogene with some synapse membrane receptors in the DG of OVX rat female rats. **(A)** Oil-injected, non-SE rats; **(B)** Oil-injected SE rats; **(C)** E2-replaced, non-SE rats; **(D)** E2-replaced, SE rats. A continuous red/blue line indicates significant (*P*-value < 0.05) expression synergism/antagonism of *Jun* with the linked gene, an interrupted black line indicates significant (*p*-value < 0.05) expression independence, while missing lines mean that we have not enough evidence to categorize the expression coordination of *Jun* with that gene.

It is interesting how the expression coordination of *Jun* with major synapse membrane receptors depends on the E2 presence and changes following SE. As illustrated in Figure 14, the E2-replacement doubled the number of synergistically expressed partners for *Jun* among the synaptic receptors and turned *Gabra1* from an independently to a synergistically expressed gene. In both oil-injected and E2-replaced animals, SE induced antagonistic expression of *Jun* with several synaptic receptors.

## Discussion

We show that the well-known neuroprotective effects of E2, in this case against SE-induced hippocampal damage, involve complex, multifactorial changes including alteration of neurotransmission pathways. Our study stresses the importance of considering global genomic changes when studying effects of steroid hormones. The neuroprotective effects of E2 against SE-induced damage involve widespread regulation of neurotransmitter systems. Our data further shows that loss of estrogen leads to a state of transcriptomic instability, which may significantly contribute to neuronal vulnerability and, consequently, may render the system more susceptible to neurodegenerative processes. We identified a large number of down-regulated genes in the neurotransmission pathways induced by SE in oil-injected females and interpreted this finding as the major culprit for most of SE features. Indeed, E2 replacement led to restoration of the expression of many of these genes and we consider this as a good indication of the E2 protective role of brain circuitry.

The overall technical noise of the arrays hybridized with samples from oil-injected animals is practically equal to that of the samples from E2-replaced animals as indicated by comparing the control spots. Hence, the lower average (and median) of the FC distribution in the COS vs CON results from the reduction of expression variability in the compared conditions. This result is consistent with our robust finding in hundreds of transcriptomic experiments that the expression variability is larger in tissues of animals closer to their normal condition than in disease conditions. As presented in a previous publication (Iacobas, 2016) CES and CEN are closer to the conditions of intact females with/without SE than are COS and CEN.

The coordination analysis of the over 80 mil gene pairs in each condition, sorted in numerous interplays among the genomic fabrics of functional pathways provides an enormous amount of information. Complete presentation of this information is not possible to cover in a single report. Therefore, we have selected some results that may enhance our understanding of the complex molecular mechanisms triggered by the SE and the neuroprotective role of E2 replacement on brain circuitry.

Studies in patients with epilepsy suggest that particularly in the DG, seizure-induced hippocampal damage (sclerosis) is associated with memory deficits (Helmstaedter et al., 1997; Grunwald et al., 1999a,b). Recordings from resected human hippocampal specimens indicate that in patients with temporal lobe epilepsy (TLE) that was associated with hippocampal sclerosis, TBS-induced LTP in the DG is lost (Beck et al., 2000). Our present data show that following kainic acid-induced SE, a model of seizures originating in the temporal lobe (Velíšková et al., 2000; Velíšková and Velíšek, 2007), TBS-induced LTP is severely impaired in slices from oil-injected, but not E2-replaced OVX rats. Interestingly, an MRI study has demonstrated that young women are less likely to develop TLE-associated hippocampal damage suggesting possible protective effects of female ovarian hormones (Briellmann et al., 2000). Indeed, our previous studies showed that kainic-acid-induced SE was associated with severe neuronal loss in the vulnerable hilar region of the DG in OVX rats but the neuronal damage was significantly ameliorated in E2-replaced animals (Velíšková et al., 2000; Velíšková and Velíšek, 2007). We would like to emphasize that in OVX rats, the dose of E2 used in our studies produces circulating levels of E2 within the physiological range (Neal-Perry et al., 2005). This E2 dose is not only neuroprotective against the SE-induced hippocampal damage but it also has a mild anticonvulsant effect (Velíšková et al., 2000; Velíšková and Velíšek, 2007). Others have used various doses of E2 and showed consistent neuroprotective effects against seizure-induced hippocampal neuronal damage regardless the E2 dose but the supraphysiological E2 levels were associated with a proconvulsant effect (see Velíšková and DeSantis, 2013 for detailed review).

Very important findings of this study concern Jun oncogene (*Jun*) expression regulation and coordination with neurotransmission genes. As part of the activator protein-1 (AP-1), *Jun* is involved in major cellular processes including differentiation, proliferation and cell survival, frequently leading to cancerous processes, (Papoudou-Bai et al., 2017; Shrihari, 2017; Tewari et al., 2017). Significant up-regulation of *Jun* by SE in oil-injected rats (2.9x) can explain vast occurrence of SE-related brain damage through activation of pro-apoptotic genes (Kravchick et al., 2016). Importantly, expression of *Jun* was not altered by SE in E2-replaced animals, proving again the important protection E2 provides to the brain likely through its interference with apoptotic process here represented by *Jun*.

In a recent study (Iacobas et al., 2018) on a rat model of infantile spasms, we have analyzed how the epileptic spasms alter the neurotransmission in the hypothalamic arcuate nucleus and how the ACTH or PMX53 (a potent complement C5ar1 antagonist) treatment recovers most of these alterations. Thus, the question exists whether initiation of the E2 replacement in adult OVX females following the SE would also rescue the deteriorating effects of SE.

## Author contributions

DI designed the microarray experiment, developed the analytical methods, and analyzed the data. SI performed the microarray experiment and the primary analysis. NN performed most of the animal work and analyzed the electrophysiology LTP data. LV contributed the biological interpretation of the experimental results. JV designed and supervised the entire project. All authors contributed to the manuscript writing.

### Conflict of interest statement

The authors declare that the research was conducted in the absence of any commercial or financial relationships that could be construed as a potential conflict of interest.

## Supplementary material

Supplementary Table S1 Significantly regulated genes in the analyzed pathways. COS/CON, CES/CEN = expression ratios (negative for down-regulation) in the respective comparisons, CUT = fold-change cut-off computed for each gene in each comparison to be considered as regulated. Red/green background of the expression ratio indicates significant up/down regulation while yellow background indicates not significant regulation.

## Abbreviations

5HT: Serotonergic synapse pathway
ACH: cholinergic synapse pathway
ALZ: Alzheimer's disease pathway
APO: apoptosis pathway
CC: cell cycle pathway
CG: common genes
CUT: absolute fold-change cut-off to consider a gene as regulated
DA: dopaminergic synapse pathway
DG: dentate gyrus
E2: β-estradiol
ESG: estrogen signaling pathway
FC: fold-change
GABA: GABAergic synapse pathway
GLU: glutamatergic synapse pathway
KA: kainic acid
LTD: long-term depression pathway
LTP: long-term potentiation pathway
OVX: ovariectomized
PPR: Pathway Protection
SE: status epilepticus
SVC: synaptic vesicle cycle pathway
TBS: theta burst stimulation
TLE: temporal lobe epilepsy
TPR: Transcriptomic Protection
WPR: Weighted Pathway Regulation
